# Distinct impacts of the 1997–98 and 2015–16 extreme El Niños on Japanese eel larval catch

**DOI:** 10.1038/s41598-018-37569-5

**Published:** 2019-02-04

**Authors:** Yong-Fu Lin, Chau-Ron Wu

**Affiliations:** Department of Earth Sciences, National Taiwan Normal University, Taipei, Taiwan

## Abstract

Extraordinarily poor recruitment of Japanese eels in East Asia has been generally reported during extreme El Niño years. However, the scenario failed to take place during the 2015–16 extreme event. In this study, we examined possible factors responsible for differing eel abundance in East Asia during the two strongest recent extreme El Niños, which occurred in 1997–98 and 2015–16. Numerical tracer experiments were carried out to determine why the impacts on eel catches seen in 1997–98 were not repeated in 2015–16. Among physical factors, two scenarios are likely responsible for extremely poor recruitment in East Asia: southward migration of the North Equatorial Current (NEC) or southward movement of eel spawning grounds. Comparing the latitudinal shift of NEC locations between the 1997–98 and 2015–16 El Niños, we conclude that NEC migration may be a factor, but is not chiefly responsible, for lower eel catches. Our findings pointed to southward movement of spawning grounds as the dominant factor. The northward movement of spawning grounds during 2015–16 meant that eel larvae were preferentially transported into the NEC-Kuroshio system, which resulted in a higher rate of recruitment success. The distinct evolution and dynamics of these two El Niño events led to different spawning ground locations, impacting eel abundance in East Asian countries.

## Introduction

**Figure 1:**
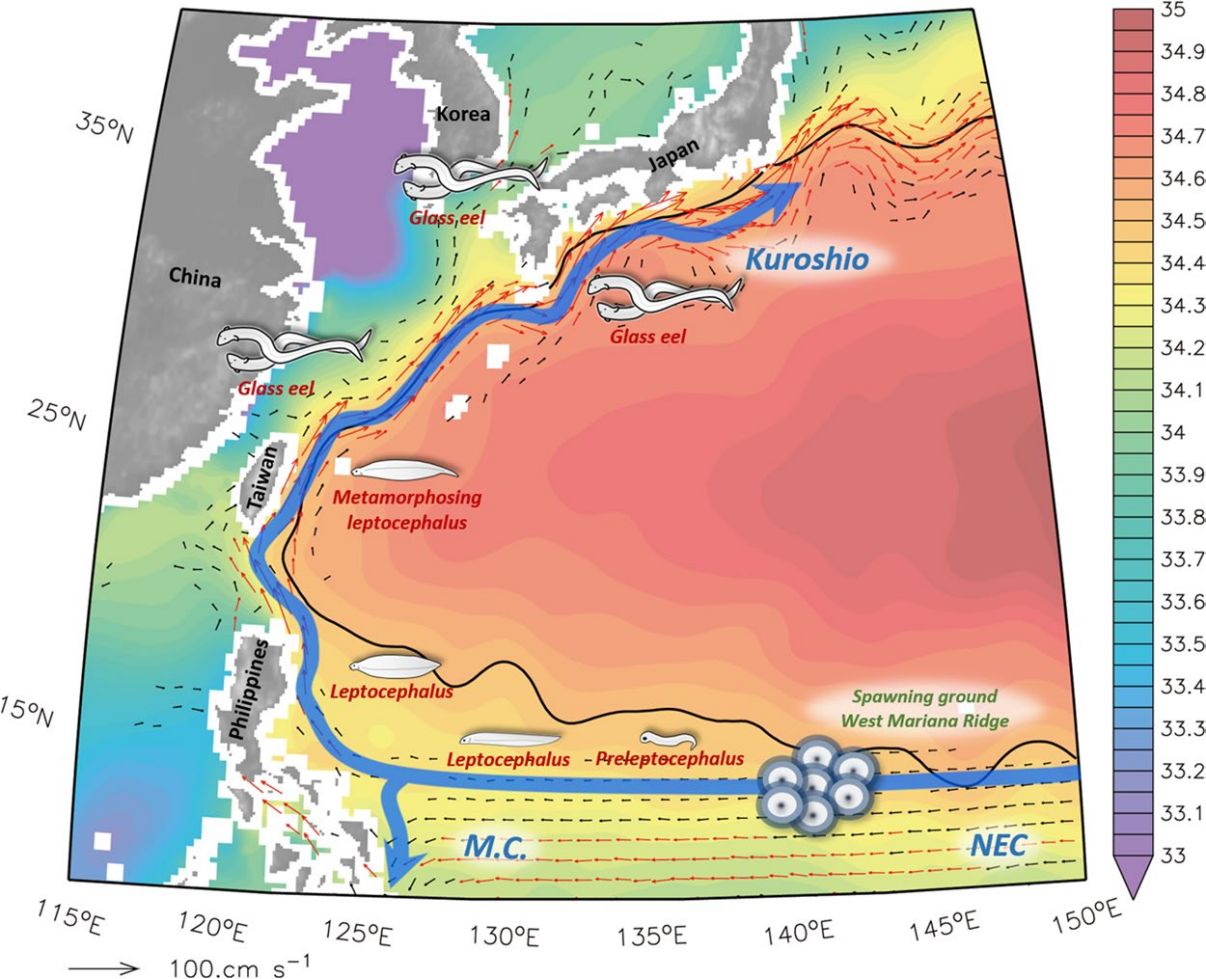
Biogeographic distribution of Japanese eels in the northwestern Pacific Ocean. Blue arrows are the schematic circulation pattern for the Kuroshio, North Equatorial Current (NEC), and Mindanao Current (M.C.). Colored shadings are annual mean of SSS averaged over depths of 0~50 m based on WOA13 (unit in PSU), and thin black curve denotes the salinity front (34.5 PSU). Black and red arrows indicate current velocities with speeds of 10~20 cm s^−1^ and >20 cm s^−1^, respectively. This figure is generated with FERRET (v6.93; http://ferret.pmel.noaa.gov/Ferret) and Microsoft Power point 2013, and refers to Shinoda *et al*.^12^.

Japanese eels traverse thousands of kilometers over several months to spawn around 12–15°N, south of the salinity front in the North Pacific (34.5 PSU)^1–4^. Eel larvae in their early life stages are poor swimmers and fundamentally carried by ocean currents, especially the North Equatorial Current (NEC) and Kuroshio, to their freshwater habitat (Fig. 1). Recent studies have demonstrated that the abundance of Japanese eels in East Asian countries has significantly declined during the recent decades^5^. Climate variability and the associated ocean current fluctuations are primary factors driving the extraordinarily poor recruitment of the Japanese eel^2,6,7^. For example, Zenimoto *et al*.^6^ showed that fewer particles (eel larvae) were carried by ocean currents during El Niño years. Kimura *et al*.^2^ further found that extraordinarily reduced eel catches in East Asian countries usually occur during the strongest El Niño events, such as the 1997–98 extreme event.

**Figure 2:**
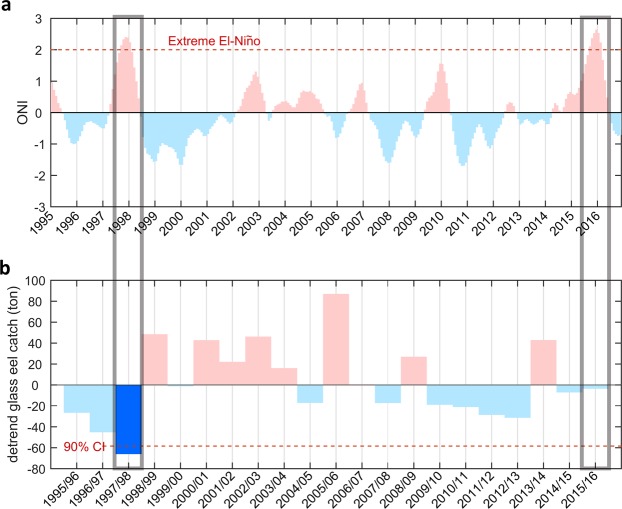
ONI indices and annual glass eel catch. (**a**) Time series of the ONI index. Positive (negative) ONI indices are shown in red (blue) colored bars. (**b**) Annual glass eel catch data for the entire fishing season in East Asia (Taiwan, China, Korea, and Japan) during the period from 1995 to 2015. Data were gathered from the Japan Aquaculture Information News (The Nihon Yoshoku Shimbun, Tokyo, Japan). Multiple year trend has been removed. A significant low eel catch has been found in 1997–98 (dark blue colored bar). Gray boxes indicate the 1997/98 and 2015/16 extreme El Niños. The figures are generated with Matlab (R2015a; https://www.mathworks.com/).

Comparable to the 1997–98 El Niño event, the recent extreme El Niño of 2015–16 has a similar strength (Fig. 2a), with maximum sea surface temperature (SST) anomalies reaching ~2.5 °C. The evolution of these two events was also apparently similar, although they started to diverge in the decaying phase. Therefore, we asked whether the 2015–16 extreme El Niño resulted in lower eel catches, similar to previous extreme El Niños.

Figure 2b shows annual glass eel catch data with the linear trend removed for the entire fishing season in East Asian countries (Japan, Korea, China, and Taiwan) during the period from 1995 to 2015. Extremely poor recruitment was observed in 1997–98, but not in the most recent extreme El Niño of 2015–16; this is against intuitive knowledge that the two extreme El Niños are similar. The reason that the impact of the 1997–98 extreme event on eel catches in East Asian countries was not repeated during the 2015–16 event merits further investigation.

## Physical Factors Responsible for Poor eel Catches

Among physical factors, collective wisdom points to two factors most likely responsible for extremely poor Japanese eel recruitment in East Asia: southward migration of the NEC^8^ or southward movement of eel spawning grounds^7^. Chang *et al*.^8^ suggested that spatial variation of the NEC is an important determinant of the distribution and migration of eel larvae. A northward-shifted NEC is capable of covering almost the entire spawning ground (about 12–15°N) and transporting a large number of eel larvae into the Kuroshio, which may increase eel catches in East Asian countries. In contrast, eel larvae cannot be transported by the NEC when it migrates southward, resulting in smaller eel catches. Through comparison of the latitudinal shift of NEC locations between the 1997–98 and 2015–16 El Niños, we conclude that southward migration of the NEC is not solely responsible for differences in eel recruitment, as demonstrated previously^8^.

The variability of the NEC bifurcation latitude (NECBL) off the Philippines is a useful proxy for meridional migration of the NEC. NECBL variability was compared between the 1997–98 and 2015–16 El Niños (Fig. S1). During the eel spawning season (May–July), the NECBL in 1997–98 was shifted farther north than in 2015–16. This suggests that the NEC in 1997–98 extended to cover most of the spawning grounds, and eel larvae were capable of reaching the nursery grounds via the NEC and Kuroshio, in contrast to 2015–16. However, markedly reduced eel catches during the 1997–98 El Niño, as shown in Fig. 2b, indicates that meridional migration of the NEC is not chiefly responsible for the reduced eel catch.

**Figure 3:**
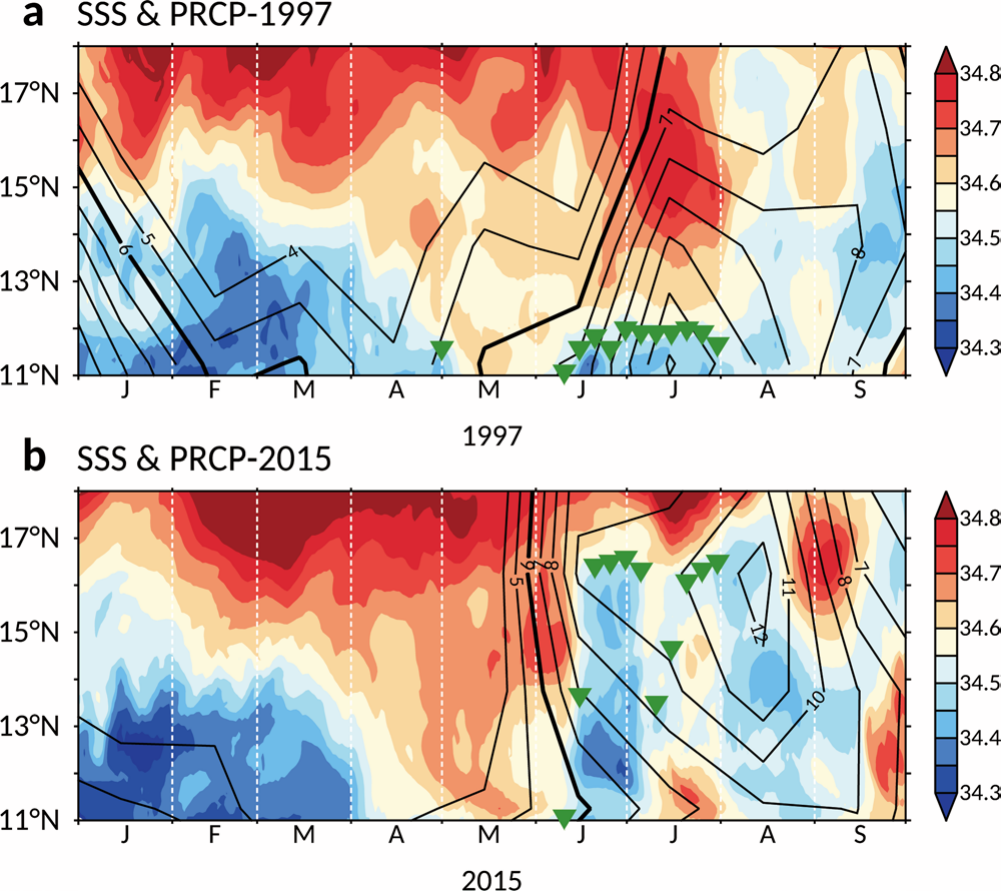
Correlation between precipitation and SSS. (**a**) SSS and precipitation averaged along 140°E during the 1997–98 El Niño event. Colored shading denotes SSS (unit: PSU), while contour is precipitation (unit: mm/day). The solid contour denotes the high rainfall zone (>6 mm/day). Contour interval is 1. Green triangles indicate possible spawning grounds (PSG) associated with meridional displacements of the salinity front (34.5 PSU). (**b**) Same as (**a**), but for the 2015–16 El Niño event. The figures are generated with FERRET (v7.4; http://ferret.pmel.noaa.gov/Ferret).

Aside from migration of the NEC itself, meridional migration of the salinity front has been demonstrated to impact eel abundance in East Asian countries^6,7,9^. The location of the salinity front identified by the sea surface salinity (SSS) pattern is chiefly associated with the precipitation distribution in the equatorial Pacific Ocean. Previous studies have suggested that spawning grounds of Japanese eels are not confined to a fixed latitude, but are instead modulated by the location of the salinity front^2,4,10^. Figure 3 shows SSS and precipitation averaged along 140°E, as well as time-series meridional displacement of the salinity front (denoting possible spawning grounds) during the 1997 and 2015 events. During the eel spawning season, the high-rainfall zone (>6 mm/day) extended farther south in 1997 (Fig. 3a) than in 2015 (Fig. 3b). Thus, the eel spawning grounds that accompany the salinity front extended farther south beyond the NEC in 1997 (south of 11°N, Fig. 3a), and newly hatched larvae cannot be successfully carried by the NEC, leading to extraordinarily diminished eel catches in East Asian countries. On the other hand, there was high precipitation after June in 2015, expanding the fresher water zone and thus causing enhanced northward migration of the salinity front (although the salinity front was situated south of 11°N by early June; Fig. 3b). Northward movement of eel spawning grounds is favorable for transport of eel larvae into the NEC-Kuroshio system, resulting in greater eel catches during the 2015–16 El Niño.

We conducted two simulated tracer experiments with the same ocean currents to better understand the effect of a latitudinal shift in spawning locations on the recruitment success of the Japanese eel. These tracer experiments were based on a data-assimilation ocean model (JCOPE2). Experiment 1 (EX1) was carried out using the predetermined locations of eel spawning grounds in 1997, with the particles (v-larvae) released from locations spread throughout the region of 140 to 142°E along 12°N, as shown in Fig. 3a, with a separation distance of 10 km in both the meridional and zonal directions and one particle released from each location. The releasing time was over a span of three months starting on May 1 to the end of July, the main spawning season of the Japanese eel^1,11,12^, and each release was staggered at a 5-day interval. Approximately 1,350 v-larvae were released each year.

**Figure 4:**
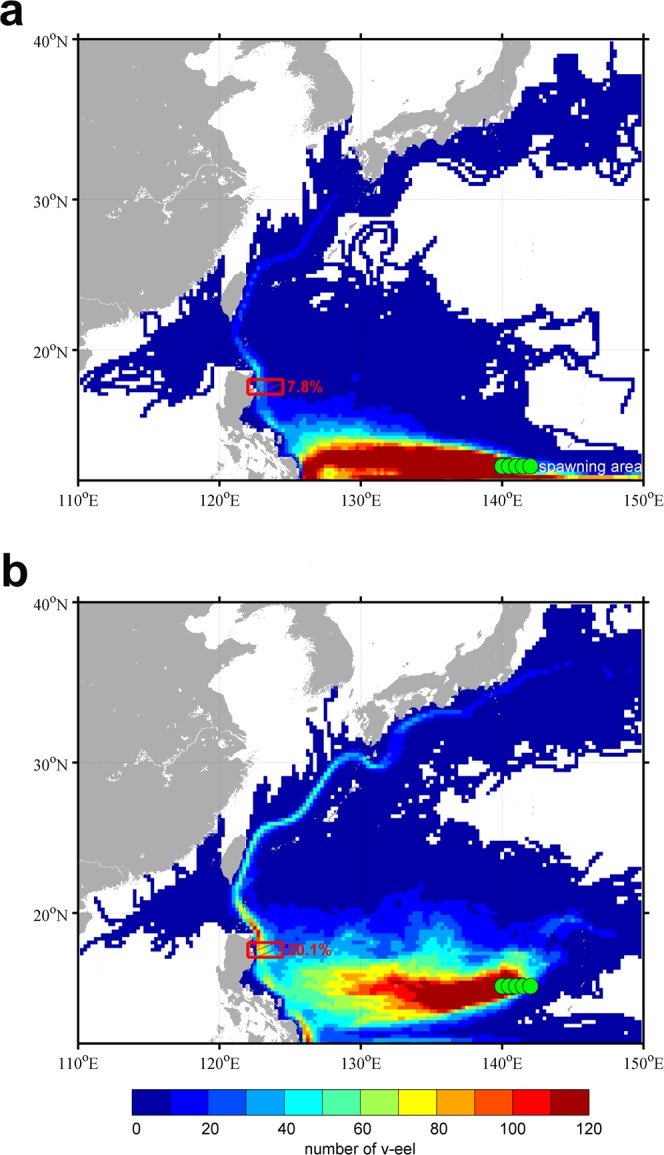
Simulated tracer trajectories. (**a**) Trajectories from a fixed spawning point of 12°N from 240-day simulation. Color scale shows cumulative drift number. (**b**) Same as (**a**), but trajectories from a fixed spawning point of 16°N. Percentage of particles that passed the red frame of the Kuroshio (17–18°N and 122–124.5°E) was regarded as the successful recruitment rate. The figures are generated with Matlab (R2015a; https://www.mathworks.com/).

The simulated particles (eel larvae) were carried by the NEC to the eastern Philippine coast, where most of the particles were then transported into the southward-flowing Mindanao Current (MC); only a few (7.8%) entered the northward-flowing Kuroshio (through the area of 17 to 18°N and 122 to 124.5°E, indicated by the red frame in Fig. 4a). The proportion for particle passing through the red frame of the Kuroshio represented the successful rate of eel recruitment. Thus, eel larvae may be preferably transported into the MC, but not the Kuroshio, resulting in relatively poor recruitment in East Asian countries during the 1997–98 extreme El Niño.

The design of experiment 2 (EX2) matched that of EX1, except it was carried out using the predetermined locations of eel spawning grounds in 2015. The v-larvae were released at locations spread through the region of 140 to 142°E along 16°N, as shown in Fig. 3b. Figure 4b shows the tracer trajectories from EX2. The v-larvae were spread latitudinally over the westward-flowing NEC main stream between 12 and 15°N, and favorable for eel larvae being delivered into the Kuroshio rather than the MC as they arrived at the eastern Philippine coast. Thus, the eel larvae were preferentially transported into the Kuroshio with a higher successful recruitment rate in EX2 (18.4%), resulting in relatively high recruitment in East Asia.

## Distinct dynamics of the two types of El Niño

**Figure 5:**
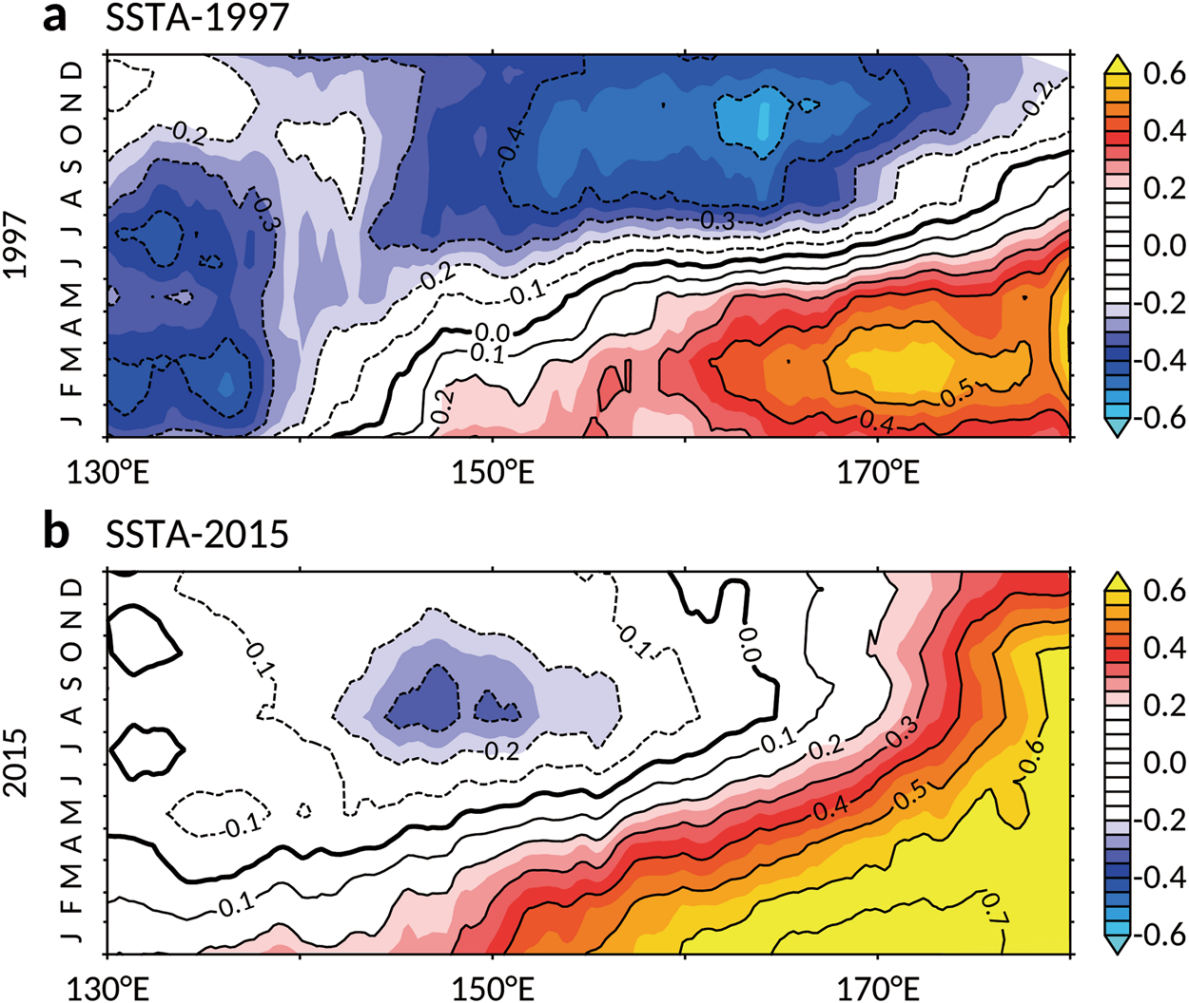
Different SSTA distribution between the 1997–98 and 2015–16 El Niños. (**a**) SST anomaly averaged 0–15°N with 5-month running mean for the 1997–98 El Niño event based on AVHRR OISST data. Colored shadings and contours denote the SST anomalies (SSTA, unit: °C). Contour interval is 0.1. (**b**) Same as (**a**), but for the 2015–16 El Niño event. The figures are generated with FERRET (v7.4; http://ferret.pmel.noaa.gov/Ferret).

SST distribution is highly related to the precipitation pattern in the tropical ocean since high rainfall generally develops in the high SST regions, where latent heat is released in organized deep convection^13,14^. To further investigate SST distribution in the west Pacific warm pool, composites were created for the extreme El Niños of 1997–98 and 2015–16 (Fig. 5). Dramatic cooling in the northwestern Pacific is observed during the eel spawning season of 1997–98 (Fig. 5a), and this cooling is approximately three-fold greater than that in 2015–16 (Fig. 5b). This difference indicates that the warm pool migrated farther east during the 1997–98 El Niño, resulting in lower rainfall in the western equatorial Pacific, as shown in Fig. 3a. On the other hand, as the warm pool maintained its position during the 2015–16 El Niño (Fig. 5b), heavier rainfall occurred over the spawning grounds (Fig. 3b), in contrast to the 1997–98 El Niño.

The distinct SST anomalies of the 1997–98 and 2015–16 El Niños indicate that the two strongest El Niño events differed greatly in the light of their underlying dynamics and associated climate influences. The 1997–98 extreme event is representative of the canonical Eastern-Pacific El Niño (EP-El Niño)^15^, while the 2015–16 extreme event is a blend of the Central-Pacific (CP) and EP El Niño types, where the distinguishing characteristic of CP-El Niño affected the 2015–16 event and made it distinct from the 1997–98 event^16^. In 1997–98, the anomalous slope of the equatorial thermocline was set up rapidly and was proportional to the wind stress^17^, resulting in a deeper thermocline and warming in the eastern equatorial Pacific, along with a shallower thermocline and cooling in the western equatorial Pacific. On the other hand, the strong CP-El Niño dynamics in 2015–16 sustained large positive SST anomalies in the central Pacific^16^, and thus no intense cooling of the warm pool occurred during the 2015–16 event. Furthermore, the SST anomaly in the Pacific warm pool preceded the precipitation anomaly by 0–4 months^7^, and the precipitation distribution indicated that the location of the salinity front is an indication of possible spawning grounds. The distinct evolution and dynamics of the two types of El Niño (i.e., EP and CP El Niño) led to differing locations of eel spawning grounds, impacting eel abundance in East Asian countries.

## Conclusion

**Figure 6:**
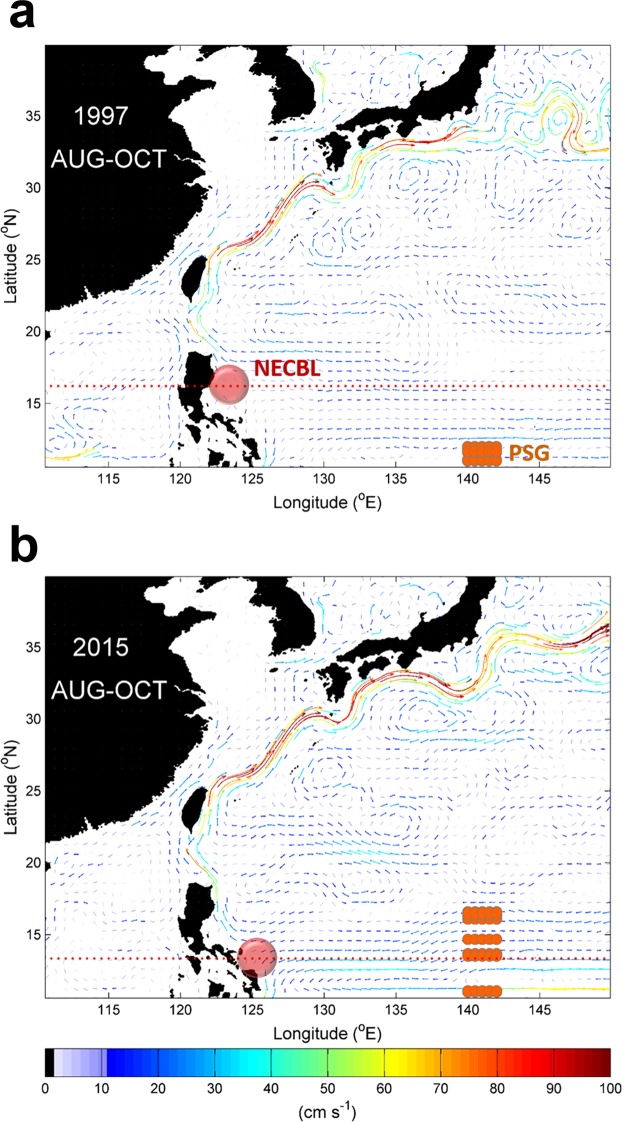
Circulation pattern between the 1997–98 and 2015–16 El Niños. Vertically averaged current speed in the top 200 m from August to October of (**a**) the 1997–98 El Niño and (**b**) the 2015–16 El Niño, calculated from the JCOPE2 product. Color scale shows the current speed. Orange rectangles denote possible spawning grounds (PSG). Red circle indicates the NECBL. The figures are generated with Matlab (R2015a; https://www.mathworks.com/).

The 2015–16 extreme El Niño did not result in extremely poor recruitment of Japanese eel in East Asia, as have other extreme El Niños. Rather than southward migration of the NEC, the present study demonstrated that southward movement of eel spawning grounds was mainly responsible for lower eel catches. During the eel spawning season (May–July) of 1997–98, Japanese eels spawned beyond the range of the NEC as the salinity front was shifted to a lower latitude (south of 12°N). The eel larvae drifted to the eastern coast of the Philippines around August-October when the NCEBL reached its northernmost position (~16°N). The larvae consequently were not capable of being successfully carried by ocean currents (the NEC and Kuroshio), which resulted in extraordinarily diminished eel catches in East Asia (as shown in Fig. 6a). On the other hand, significant northward movement of the salinity front (denoting the eel spawning grounds) occurred after June in 2015. The northward movement of eel spawning grounds is favorable for transport of larvae into the NEC and Kuroshio, resulting in higher eel catches during the 2015–16 El Niño (Fig. 6b).

The dissimilar underlying dynamics of the 1997–98 and 2015–16 extreme El Niño events led to different SST anomalies and associated precipitation patterns in the northwestern Pacific. The changing location of eel spawning grounds was accompanied by latitudinal migration of the salinity front, resulting from distinct precipitation distributions associated with the two El Niños.

## Methods

### Data sets

- *Annual glass eel fishing data* in East Asian countries (Japan, Korea, China, and Taiwan) during the period from 1995 to 2015 are gathered from the Japan Aquaculture Information News (The Nihon Yoshoku Shimbun, Tokyo, Japan).
- *Ocean surface velocity data* are adopted from Satellite Oceanographic data (AVISO version DT-MADT and DT-MSLA, two sat merged of Ssalto/Duacs, http://www.aviso.altimetry.fr). The AVISO data include sea surface height anomalies and surface geostrophic current velocities that are gridded on a horizontal region of 0.25 degree with an interval of one day.
- *Salinity data* are adopted from World Ocean Atlas 2013 version 2 (WOA13v2, https://www.nodc.noaa.gov/about/oceanclimate.html) and Japan Coastal Ocean Predictability Experiment 2 (JCOPE2, http://www.jamstec.go.jp/e/)^18^. The WOA13 dataset is based on *in-situ* measurements in climatological fields on global grids (0.25° × 0.25°) and 102 vertical layers. The JCOPE2 model domain covers the western North Pacific Ocean from 10.5°N to 62°N latitude and 108°E to 180°E longitude with a resolution of 1/12° in horizontal and 46 sigma levels in vertical.
- *The* 1/4° *daily SST data* are the product of the National Oceanic and Atmospheric Administration (NOAA) Optimum Interpolation Sea Surface Temperature (OISST) version 2 (http://www.ncdc.noaa.gov/oisst), which is distributed by NOAA’s National Climatic Data Center (NCDC).
- *Precipitation data* are adopted from the version 2.2 of the Global Precipitation Climatology Project (GPCP V2.2). The product combines *in-situ* measurements and satellite precipitation data into 2.5° × 2.5° global grids^19^. Monthly averages since 1979 are available at the Physical Sciences Division (PSD) of the NOAA’s Earth System Research Laboratory (NOAA/ESRL, http://www.esrl.noaa.gov/psd/data/gridded/data.gpcp.html).

### Tracer experiments

The tracer experiments were executed to gain an understanding of the possible passive behavior of eel larvae, and based on a data-assimilation ocean model (known as JCOPE2). The JCOPE2 model domain covered the western North Pacific Ocean from 10.5°N to 62°N latitude and 108°E to 180°E longitude with a resolution of 1/12° in horizontal and 46 sigma levels in vertical. The formulation of the JCOPE2 followed that of the sigma-coordinate Princeton Ocean Model (POM)^20^. *In-situ* measurements of temperature and salinity, together with satellite remote sensing data were assimilated into the JCOPE2 model^18^.

Since the eel spawning area was particularly located between 12°N and 15°N and along the western edge of the West Mariana Ridge^1^ (about 140–142°E), the particles (tracers) were released at locations spread over the region of 140°E to 142°E, along either 12°N (1997) or 16°N (2015). Furthermore, Eel larvae likely exhibit diel vertical migration (DVM)^21^ behavior, remaining in the surface layer at night and swim to a deeper layer during the daytime. In this study, thus, the particles were set to stay at a fixed depth of 50 m during the nighttime, and a fixed depth of 150 m during the daytime. The releasing time was over a span of three months starting on May 1 to the end of July and each release was staggered at a 5-day interval.

## Supplementary information

Supplementary Info
