# Supplementary material for: Distinct impacts of the 1997–98 and 2015–16 extreme El Niños on Japanese eel larval catch
Source: Sci Rep. 2019 Feb 4;9:1384. doi: 10.1038/s41598-018-37569-5 (PMC6361929; doi:10.1038/s41598-018-37569-5)
Supplement: Supplementary file 1 — Supplementary Info [file 41598_2018_37569_MOESM1_ESM.docx]

**Supporting Online Material for:**

**Distinct impacts of the 1997–98 and 2015–16** **extreme El Niños on Japanese eel larval catch**

Yong-Fu Lin^1^ and Chau-Ron Wu^1,*^

^1^Department of Earth Sciences, National Taiwan Normal University, Taiwan

*Correspondence to cwu@ntnu.edu.tw

**Variability of the NEC bifurcation latitude between the 1997–98 and 2015–16 El Niños**

The variability of the NEC bifurcation latitude (NECBL) off the Philippines is served as a good proxy for the meridional migration of the NEC. Figure S1 compares the NECBL variability between the 1997–98 and 2015–16 El Niños.


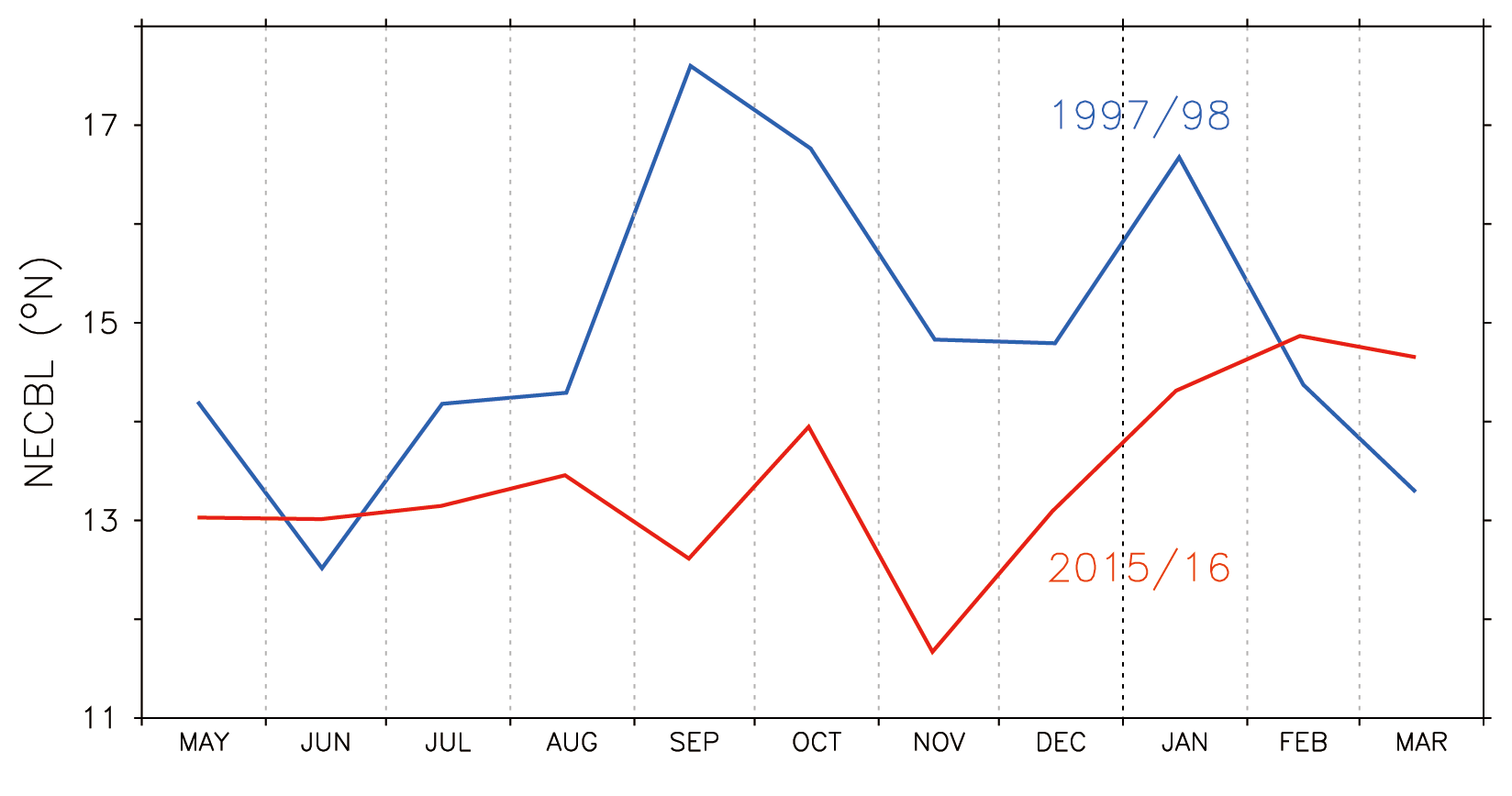


Figure S1. The NECBL variability of (a) the 1997–98 and (b) 2015–16 El Niño events, calculated from the altimeter-based sea level anomaly by AVISO. The figure is generated with FERRET (v7.4; http://ferret.pmel.noaa.gov/Ferret).

**Datasets**

*Sea level anomaly (SLA) data* are adopted from Satellite Oceanographic data (AVISO version DT-MADT and DT-MSLA, two sat merged of Ssalto/Duacs, <http://www.aviso.altimetry.fr>). The AVISO data provide surface geostrophic velocities and sea level anomalies that are sampled daily with a spatial resolution of 0.25°. The NEC bifurcation latitude (NECBL) is calculated from monthly AVISO data ^1^. The meridional geostrophic velocity ($v_{g}$) is first calculated as a function of y (latitude) along the Philippine coast (averaged in a 1-degree band off east Philippine). For each month, NECBL is obtained where$v_{g}(y, t)=0$.

**References**

1. Qiu, B., & Chen, S. [Interannual-to-decadal variability in the bifurcation of the North Equatorial Current off the Philippines.](http://www.soest.hawaii.edu/oceanography/bo/QC_NEC.pdf) *J. Phys. Oceanogr.* **40**, 2525-2538 (2010).
